# The Relationship of Internet Abusive Use with Academic Burnout and Academic Performance in Nursing Students

**DOI:** 10.1155/2022/2765763

**Published:** 2022-04-05

**Authors:** Faranak Jafari, Maryam Janatolmakan, Safura Khubdast, Seyyed Mohsen Azizi, Alireza Khatony

**Affiliations:** ^1^Student Research Committee, Kermanshah University of Medical Sciences, Kermanshah, Iran; ^2^Social Development and Health Promotion Research Center, Health Institute, Kermanshah University of Medical Sciences, Kermanshah, Iran; ^3^Medical Education and Development Center, Arak University of Medical Sciences, Arak, Iran; ^4^Infectious Diseases Research Center, Kermanshah University of Medical Sciences, Kermanshah, Iran

## Abstract

**Materials and Methods:**

A total of 184 nursing students were recruited by simple random sampling. Data collection tools included a demographic information form, the Internet Abusive Use Questionnaire and Maslach Burnout Inventory-Student Survey. Data were analyzed by the SPSS-18 software using descriptive and inferential statistics.

**Results:**

The abusive use of the Internet in nursing students was lower than the average level. There was a positive correlation between Internet abusive use and academic burnout (*r* = 0.305, *p* < 0.001), but there was a negative correlation between Internet abusive use and academic performance (*r* = −0.478, *p* < 0.001). The results of multiple regression analysis showed that Internet abusive use could predict the variance of academic burnout and academic performance.

**Conclusions:**

Internet abusive use was correlated with increased academic burnout and decreased academic performance in nursing students. Educational interventions are required to increase students' awareness of the consequences of Internet abusive use.

## 1. Introduction

Internet addiction is known as a psychosocial disorder and is included in the Diagnostic and Statistical Manual of Mental Disorders (DSM) framework [1, 2]. In addition to the concept of Internet addiction, researchers use other concepts such as pathological use of the Internet, Internet dependence, problematic use of the Internet, and Internet abuse use. Internet addiction includes various forms of social media addiction, virtual chat, online shopping, online game addiction, and overdownloading movies, photos, or apps [3, 4]. The concept of Internet abusive use refers to the circumstances in which a person uses the Internet beyond what he or she has planned [5, 6]. Those who are addicted to the Internet have low skills in managing the time to use the Internet. In addition, their quality of life and self-esteem are low [7, 8]. They also undergo high psychological damage [8].

Iran is one of the countries where the Internet has grown significantly in recent years. The results of a meta-analysis showed that the prevalence of Internet addiction in Iran was moderate [9]. According to the results of another meta-analysis, 2.1% of Iranian students were at risk of Internet addiction, and 5.3% of them were addicted to the Internet [10]. Therefore, Iranian students, like students in other countries, are exposed to the consequences of Internet addiction. Since students are among the most important Internet users around the world, dependence on and excessive use of the Internet can disrupt their academic life and social relationships [11]. Azizi et al. showed that addiction to social networks has a negative and significant effect on students' academic performance [12]. The results of a study by Craparo et al. indicated a significantly negative relationship between Internet addiction and self-efficacy in managing negative emotions [13]. The results of the study of Sharma and Sharma indicated a significantly negative relationship between Internet addiction and mental health in students. According to this study, students with high levels of Internet addiction have low psychological health [14]. Kumar et al. concluded that Internet addiction had a negative effect on students' mental health and academic performance [15]. The results of Budak et al. showed a significantly positive relationship between Internet addiction and psychological disorders [8].

One of the variables that adversely affects students' academic life is academic burnout [16]. Academic burnout indicates students' feeling of tiredness due to study requirements and difficulties, lack of interest in homework, and a sense of inefficiency [17]. Academic burnout includes three components: emotional exhaustion, cynicism, and reduced academic efficacy [18]. Evidence suggests that academic burnout is a common problem among nursing students [16, 19] and has a negative effect on their relationships, empathy, mental health [20], and academic performance [18].

Peterka-Bonetta et al. [11] reported a significant relationship between Internet use disorder and burnout in German and Chinese students [11]. Imani et al. also reported a positive correlation between Internet addiction and burnout [21]. Therefore, it is important to pay attention to the prevalence of Internet addiction and academic burnout among students.

Numerous studies have investigated the correlation between Internet addiction and academic performance in students [15, 22, 23]. However, few studies have examined the relationship between Internet abusive use and academic burnout [21]. Nursing students are one of the most important parts of the health system. Evidence shows that nursing students who are heavily dependent on the Internet face academic problems [24, 25]. They also experience high levels of stress, which may affect their ability to communicate healthily with others [26]. If nursing students perform academically well, they can be more efficient in healthcare settings. However, the prevalence of Internet addiction and other technologies such as smartphones among students has turned into a matter of concern. Therefore, studying the dimensions of this phenomenon and its effects on the academic performance of nursing students can help reduce its consequences [24]. Nursing students are one of the most important parts of the health system. Evidence shows that nursing students who are heavily dependent on the Internet have academic difficulties [24, 25]. They also experience high levels of stress, which may affect their ability to communicate healthily with others [26]. If nursing students perform academically well, they can be more effective in health care settings. However, the prevalence of Internet addiction and other technologies such as smartphones among students has become a matter of concern. Therefore, studying the dimensions of this phenomenon and its effects on the academic performance of nursing students can help reduce its consequences [24].

Therefore, this study was conducted to investigate the Internet abusive use in nursing students and its correlation with their academic burnout and academic performance. This study sought to answer the following questions:

1. What is the level of Internet abusive use in nursing students?
2. What is the relationship between Internet abusive use and academic burnout and academic performance?

## 2. Materials and Methods

### 2.1. Study Design

This cross-sectional and analytical study was conducted according to the Strengthening The Reporting of Observational Studies in Epidemiology (STROBE) guidelines.

### 2.2. Sample and Sampling Method

The study population included nursing students studying in the second or higher semesters in the academic year 2019-2020. The sample size was determined using the correlation coefficient formula of 121 people (correlation coefficient = 0.210; confidence level = 99%; test power = 0.99%), which was increased to 200 people in order to increase the accuracy of the results. The samples were recruited by the simple random sampling method. The inclusion criteria were studying in the second or higher semesters and consent to participate in the study. The reason for choosing second and higher semester students was that the academic performance of first semester students is not clear, so it is necessary to pass at least one semester. The exclusion criteria were unwillingness to participate in the study and incomplete completion of questionnaires. A total of 16 students were excluded due to incomplete completion of the questionnaires; hence, 184 students were included in the study.

### 2.3. Instruments

A demographic information form, the Internet Abusive Use Questionnaire (IAUQ) and Maslach Burnout Inventory-Student Survey (MBI-SS) were used to collect data.

The demographic information form contained 3 questions about gender, age, and grade point average (GPA) of the previous semester.

The IAUQ was designed by Calvo-Frances [5]. The internal consistency and construct validity of IAUQ have been confirmed by Calvo-Frances [5]. The Persian version of the IAUQ has been psychoanalyzed by Mottaghi and Safaie [27]. Cronbach's alpha coefficient and convergent validity have been reported to be 0.91 and 0.82, respectively [27]. In the present study, the internal consistency of the questionnaire was evaluated by Cronbach's alpha (0.886). This questionnaire has 12 items and 2 components, including functional disorder and excitability. The questionnaire is scored on a five-point Likert scale, from 0 (strongly disagree) to 4 (strongly agree). The score range of the questionnaire is between 0 and 48.

MBI-SS was designed by Schaufeli et al. [28]. The internal consistency of this questionnaire has been confirmed by previous studies [29–31]. The Persian version of MBI-SS has been standardized in Iranian students, and its reliability has been reported to be between 0.84 and 0.90 [27]. In the present study, the internal consistency of MBI-SS was examined by Cronbach's alpha (0.740). MBI-SS has 15 items and includes reduced professional efficacy (PE), emotional exhaustion (EE), and cynicism (CY). The questionnaire is graded on a seven-point Likert scale, from 0 (never) to 6 (always). The minimum score of all subscales was equal to 0. Further, the maximum score was 30 for the EX subscale, 24 for the CY subscale, and 36 for the PE subscale. Each person's score range is 0-90. MBI-SS does not have a cut-off point. Thus, a high score in the areas of “emotional exhaustion” and “cynicism” and a low score in the area of academic efficacy indicate burnout.

In order to determine the academic performance, the GPA of the previous semester was used, which has a range between 0 and 20. The GPA range is between 0 and 20. Scores less than 14 were considered poor, 14 to 17 were considered moderate, and above 17 were considered good academic performance.

### 2.4. Data Collection

Study permit was obtained from Kermanshah University of Medical Sciences (KUMS). In this regard, the researcher referred to the school of nursing based on the students' class schedule. The list of students was obtained from the department of education, and the samples were selected by simple random sampling using a table of random numbers. Then, according to their class schedule, the students were visited, and if they agreed to participate in the study, questionnaires were given to them and collected after completion. Some of the questionnaires were sent to students electronically using the Google Form tool.

### 2.5. Statistical Analysis

Data were analyzed by the SPSS software (version 18) using descriptive (frequency, mean, and standard deviation) and inferential (Pearson correlation coefficient and multivariate regression analysis) statistics.

Pearson correlation coefficient test was used to examine the correlation between the study variables. The multivariate regression analysis was used to evaluate the variance of dependent variables based on the independent variable. The level of significance for all tests was set at <0.05.

### 2.6. Ethical Considerations

The Ethics Committee of KUMS approved the study with the code IR.KUMS.REC.1400.042. The objectives of the study were explained to the participants. Informed written consent was also obtained from all participants.

## 3. Results

Out of a total of 184 students, most of the samples were female (63%, *n* = 116), undergraduate (75.5%, *n* = 138), and in the age group of 19-22 years. The mean age of the samples was 23.7 ± 4.5 years (Table 1).

The mean Internet abusive use was 30.7 ± 9.4 out of 40. Further, the mean academic burnout was 45.8 ± 9.0 out of 90 (Table 2).

Moreover, 65.2% (*n* = 120) of students used the Internet normally, and 34.8% (*n* = 64) had abusive use of the Internet. There was a positive correlation between Internet abusive use and academic burnout (*r* = 0.305, *p* < 0.001), but there was a negative correlation between Internet abusive use and academic performance (*r* = −0.478, *p* < 0.001) (Table 3).

The results of multivariate regression analysis showed that the Internet abusive use explained 0.23% and 0.10% of variance in academic performance and academic burnout, respectively (Table 4).

## 4. Discussion

This study was aimed at investigating the rate of Internet abusive use in nursing students and its correlation with their academic burnout and academic performance. The results showed that more than one-third of students had abusive use of the Internet, which was below average. The studies that have examined the prevalence of Internet addiction in Iranian students have reported a prevalence rate of 5.2%-12.9% [10, 32]. In other studies, 10.3% of Polish nursing students [33], 3.8% of Turkish nursing students [34], 0.9% of Pakistani medical undergraduates' students [35], 8.3% of Chinese adolescents [36], 17.3% Iranian nursing students [37], 42.69% and 10.31% of Saudi and Egyptian nursing students [25], and 4% of the American college students were highly addicted to the Internet [38]. In general, these statistics show that Internet addiction is a common phenomenon around the world. Differences in the level of Internet addiction or abusive use of the Internet among students in different countries can be associated with socio-cultural factors. In addition, the level of Internet access and the use of smartphones may play a role in Internet addiction [35]. Therefore, the factors and context of the development of Internet addiction should be considered in implementing educational programs for the treatment of Internet addiction.

In the present study, a significantly negative correlation was found between Internet abusive use and academic performance. This part of our results was consistent with the findings of Azizi et al. and Kumar et al. [12, 15]. In this regard, the results of a study by Fatehi et al. showed that the mean GPA was low in students with Internet addiction [7].

In this study, a positive significant correlation was found between Internet abusive use and academic burnout. This finding is consistent with the study of Peterka-Bonetta et al. [11]. Sharma and Sharma also reported that Internet addiction had a negative correlation with psychological health [14]. Mei et al. showed a correlation between problematic use of the Internet and self-control, self-esteem, and health [36]. Berte et al. also reported a negative correlation between Internet addiction and perceived self-efficacy [39].

Abusive use of the Internet can have many negative consequences for students. Academic burnout and poor academic performance are among the most important consequences of Internet abusive use. Students who are addicted to the Internet spend a lot of time on the Internet, which can prevent them from taking care of their academic affairs and consequently make them face academic difficulties. In this regard, a study indicated more than half of the students reported that their academic performance was affected by excessive use of the Internet [23].

Given the harmful consequences of Internet addiction for students, universities should take steps to prevent, manage, and treat this disorder.

The results of a systematic review suggested that one should focus on the specific skills of individuals, such as self-efficacy, self-control, emotional intelligence, interpersonal skills, social competencies, and healthy activities to prevent Internet addiction [40]. In this regard, holding training workshops and performing medical interventions are suggested.

This study had several limitations. This study was cross-sectional, so no causal explanations could be derived from it. The data collection tool was a self-report questionnaire, which might have affected the accuracy of the results. However, the researcher tried to reduce this limitation by emphasizing the confidentiality of information and anonymity of the questionnaires. Moreover, the mental state of students while completing the questionnaires as well as physiological and social-related factors might affect the results of the study, which were beyond the control of the researcher.

## 5. Conclusion

The results showed that more than one-third of Iranian students had abusive use of the Internet, which was significantly correlated with their academic performance and academic burnout. Abusive use of and dependence on the Internet can disrupt students' academic and professional lives. Therefore, the authorities should take the necessary measures to increase students' knowledge about the academic and professional risks of Internet addiction. In this regard, holding training workshops can be useful. Referral of Internet-addicted students to a psychiatrist/psychologist should also be considered. Future studies are suggested to explore the causes of Internet addiction using the causal method. In this regard, the use of qualitative methods can be useful. Similar studies are also advised to be performed on other students of health professions.

## Figures and Tables

**Table 1:** Demographic characteristics of nursing students.

Variables		*n* (%)
Sex	Male	68 (37.0)
	Female	116 (63.0)
Age (years)	19-22	96 (52.2)
	23-26	58 (31.5)
	27-30	14 (7.6)
	≥31	16 (8.7)
Education	Bachelor of science	138 (75.5)
	Master of science	46 (25.0)

**Table 2:** Mean and standard deviation of study variables.

Variables	Mean (standard deviation)	Minimum	Maximum
Internet abusive use	21.7 (9.4)	0.0	48.0
Academic burnout	45.8 (9.0)	29.0	72.0
Grade point average	16.6 (1.2)	12.0	18.7

**Table 3:** Correlation of Internet abusive use with academic burnout and grade point average.

Variable	Academic burnout		Grade point average	
	*r*	*p* value	*r*	*p* value
Internet abusive use	0.305	≤0.01	-0.478	≤0.01

**Table 4:** Results of multiple regression analysis predicting grade point average and academic burnout.

Variables	*B*	Std. error	*t*	*F*	*R* ^2^	*p* value
Internet abusive use and grade point average	-0.06	0.08	-7.34	53.92	0.23	≤0.01
Internet abusive use and academic burnout	0.29	0.06	4.31	18.58	0.10	≤0.01

Dependent variables: grade point average and academic burnout; Independent variable: Internet abusive use.

## Data Availability

The identified datasets analyzed during the current study are available from the corresponding author upon reasonable request.
